# Design and Decoding of a Novel Steering Motor Imagery Paradigm for Driving Brain–Computer Interfaces

**DOI:** 10.3390/s26185810

**Published:** 2026-09-14

**Authors:** Peiwen Mi, Zhengtong Liu, Yifei Yang, Guofeng Qin, Xin Chen, Jiajin He, Fengyi Li, Fuwu Yan, Lirong Yan

**Affiliations:** 1 The School of Automotive Engineering, Wuhan University of Technology, Wuhan 430070, China; 2 The Teachers College for Vocational and Education, Guangxi Normal University, Guilin 541004, China; 3 The School of Electronic and Information Engineering, Guangxi Normal University, Guilin 541004, China

**Keywords:** EEG, brain–computer interface, motor imagery, steering intention recognition

## Abstract

This study proposes a motor imagery brain-computer interface (MI-BCI) experimental paradigm for right-hand steering movements in driving scenarios. The proposed paradigm aligns more closely with actual steering behavior at the action level, aiming to address the mismatch between conventional motor imagery and actual driving tasks. To validate the feasibility of this paradigm, motor imagery EEG data were collected from 12 participants using an eight-electrode low-cost OpenBCI device and systematically analyzed. Time-frequency analysis revealed that, during right-hand steering imagery, electrode C3 exhibited event-related desynchronization (ERD) in both the mu and beta bands, whereas electrode C4 also showed ERD in the beta band but exhibited event-related synchronization predominantly in the mu band. Spatial pattern analysis further indicated that common spatial pattern (CSP) yielded discriminative weight distributions over the central region associated with steering direction, suggesting that the paradigm can elicit neural pattern differences corresponding to steering direction. On this basis, baseline methods, including CSP, FBCSP, EEGNet, EEG-TCNet, and ATCNet were employed to evaluate the separability of left- and right-turn steering tasks performed with the right hand under this paradigm. To improve decoding performance, the SMB-TCSAN model was developed, integrating Sinc convolution, multi-branch temporal convolution, and a multi-head self-attention mechanism, achieving an average classification accuracy of 66.80% on the steering-direction dataset. This study preliminarily validates the feasibility of low-cost, few-channel EEG devices in steering motor imagery decoding, providing a conceptual design reference for future research on driving-assistive BCI systems.

## 1. Introduction

Brain–computer interface (BCI) technology bypasses traditional muscle and neural output pathways, enabling brain signals to directly control external devices or receive feedback from external devices to the brain [1]. Non-invasive BCIs based on electroencephalography (EEG) signals have accumulated extensive research due to their high safety and convenience. They have demonstrated broad application potential in laboratory validation stages, such as brain-controlled vehicles [2], brain-controlled wheelchairs, and robotic arms [3]. Among these, motor-based BCIs decode human motor intention by identifying EEG signals associated with motor imagery, execution, or attempted movements. This capability enables the control of neural prostheses, exoskeletons, or robotic arms, thereby assisting in restoring or enhancing impaired motor functions [4,5]. With the continuous advancement of BCI technology, research on its application in the driving field is also increasing. By integrating vehicle information with the driver’s EEG signals, it is possible not only to monitor the driver’s state of consciousness but also to analyze their cognitive processes, thereby constructing more accurate models for understanding driving intention and ultimately improving vehicle driving safety [6].

EEG-based decoding of driving intention represents a significant research direction in the BCI field. Compared with electromyographic (EMG) signals, EEG signals allow earlier detection of a driver’s braking intention [7]. In the context of traffic safety, accurately recognizing driver behavior and intention is particularly crucial. Consequently, steering, the primary means of controlling lateral vehicle motion, has attracted widespread research attention. Studies indicate that when a driver performs left or right turns, event-related desynchronization (ERD) of the mu rhythm (approximately 8–13 Hz) and beta rhythm (approximately 13–30 Hz) occurs in the motor and premotor cortical areas [8]. This somatotopically organized neural activity is elicited during cognitive tasks involving motor execution, observation, and imagery [9]. Although motor imagery (MI) does not involve actual muscle contraction, it activates neural networks largely overlapping with those engaged in real movement execution, spanning regions from the premotor cortex to the cerebellum [10,11]. Building on this evidence, several studies have attempted to detect steering intention by analyzing EEG signals acquired during steering tasks. For instance, Li et al. [12] recorded EEG data in a driving simulator, extracted wavelet packet coefficient energy as features via wavelet decomposition, and fed them into a Bayesian classifier to distinguish steering from straight-driving intention, achieving a recognition accuracy of 82.6%. Similarly, Zero et al. [13] developed a BCI system that detects a driver’s intention to perform left- or right-arm movements during simulated driving using EEG signals. Together, these studies demonstrate the feasibility of EEG-based steering intention recognition.

Researchers have also developed a variety of BCI systems for steering control, utilizing paradigms such as P300, steady-state visual evoked potentials (SSVEPs), and MI. The P300 paradigm is recognized for its high accuracy and user-friendliness. For example, Lian et al. [14] developed a P300-based driving BCI capable of generating motion commands for left turns, right turns, and speed changes, achieving an accuracy of 83.59% with a detection time of approximately 2 s. SSVEP-based systems offer a high information transfer rate (ITR) and strong resistance to interference. Capitalizing on these strengths, Lu et al. [15] implemented vehicle control by projecting four flickering grid patterns onto the windshield as SSVEP stimuli. However, the paradigm’s visual fatigue poses potential risks to driving safety. The error-related potential (ErrP) is an endogenous EEG component elicited when a user perceives an error, either their own or one made by the system. BCIs incorporating ErrP can perform real-time error monitoring and self-correction, thereby improving system reliability and adaptability. In this context, Zhang et al. [16,17] developed an ErrP-based BCI designed to predict a driver’s intended turning direction before reaching an intersection, achieving an average classification accuracy of 69.8% in offline car simulator tests. Exploiting the stimulus-free nature of MI, Zhuang et al. [18] implemented a four-class MI-BCI that translates imagined movements of the left hand, right hand, tongue, and foot into steering and acceleration commands, with left- and right-hand imagery mapped to corresponding turning directions. In summary, while the aforementioned BCI paradigms can effectively recognize steering intention and enable steering control, their interaction mechanisms are inconsistent with the habitual driving behavior of actually turning the steering wheel.

The low signal-to-noise ratio (SNR), non-stationarity, and subjective variability of MI-EEG signals, compounded by variations caused by environmental and experimental factors [19], necessitate the extraction of robust features and the design of resilient recognition algorithms for the advancement of BCI applications in the domain of driving assistance. In this context, deep learning (DL) models have demonstrated considerable promise for MI-EEG classification, as they can more effectively explore and interpret complex relationships within brain physiological signals. By delivering superior classification performance and enabling automatic feature extraction, DL models are particularly advantageous for developing end-to-end BCI systems.

Convolutional neural networks (CNNs), for instance, excel at extracting local spatial features through convolutional operations. A notable example is EEGNet, a lightweight CNN architecture proposed by Lawhern et al. [20] specifically for EEG classification, which has shown strong generalization across diverse BCI paradigms. Nevertheless, the limited receptive field of standard CNNs often restricts their ability to capture long-range temporal dependencies and integrated spatiotemporal dynamics. To overcome this, Salami et al. [21] introduced EEG-ITNet, a hybrid model that combines Inception modules with temporal convolutional networks (TCNs) to effectively distill rich spectral, spatial, and temporal features from MI-EEG data. Furthermore, integrating attention mechanisms into DL architectures allows the network to adaptively emphasize task-critical signal components and suppress irrelevant noise, leading to a marked improvement in model performance. For example, Qin et al. [22] developed a network that integrates Efficient Channel Attention (ECA) with TCN, where the ECA module facilitates adaptive cross-channel interaction, improving feature discriminability in multi-channel EEG settings. In summary, deep learning frameworks built around CNNs and TCNs, particularly when augmented with attention mechanisms, have significantly advanced the capability to capture discriminative spatiotemporal patterns in MI-EEG signals, offering a robust and effective pathway toward accurate intention recognition in driving-oriented BCIs.

Decoding drivers’ steering intention via BCIs remains challenging. Both visual stimulation and MI paradigms face two major obstacles: control paradigms misaligned with natural driving, and a limited understanding of the underlying neural mechanisms. Moreover, the high cost of the medical- or research-grade sensors and computational resources hinders the widespread adoption of MI-BCI systems.

This study employed the OpenBCI device, a low-cost commercial system with a reduced number of sensors, to reduce economic and computational overhead. Inspired by natural driving habits, we designed a motor imagery paradigm based on steering wheel rotation to recognize drivers‘ left and right steering intention. To improve the decoding performance for steering intention recognition, we further proposed a CNN model termed Sinc Multi-Branch Temporal Convolutional Self-Attention Network (SMB-TCSAN) for decoding steering intention. This model serves as a dedicated tool to assess the discriminability of the EEG signals elicited by the proposed paradigm. The results provide preliminary experimental evidence for the potential of steering-based motor imagery in driving assistive BCI applications.

## 2. Materials and Methods

### 2.1. Experimental Paradigm and Procedure

A total of twelve healthy right-handed participants (10 males and 2 females, aged 23 ± 3 years) were recruited for this experiment. All participants were university students with normal or corrected-to-normal vision. Prior to the experiment, they reported no history of neurological disorders and had not consumed any alcohol, tobacco, medications, or caffeinated substances. The study was approved by the Ethics Committee of Wuhan University of Technology, and written informed consent was obtained from all participants in advance.

In routine driving scenarios, particularly during straight-line cruising, right-hand single-handed steering is relatively common. Meanwhile, right-handed individuals exhibit advantages in force control, movement precision, and motor pattern memory of the right hand, and show more stable contralateral cortical activation during right-hand motor imagery [23]. Based on these considerations, this study adopted a right-hand steering imagery task at the proof-of-concept stage to control the handedness variable, and to examine whether the paradigm can elicit directionally discriminable EEG patterns under controlled conditions.

The experiment was conducted with participants seated comfortably at a desk, facing a screen and holding a steering wheel (Logitech G29) in a standard driving posture. Prior to the formal session, the task procedure was explained to all participants, who then completed a practice session to ensure full comprehension of the instructions. During the main experiment, participants were instructed to imagine right-hand steering motions corresponding to visual cues while maintaining physical stillness and minimizing extraneous movements, including blinks.

**Figure 1:**
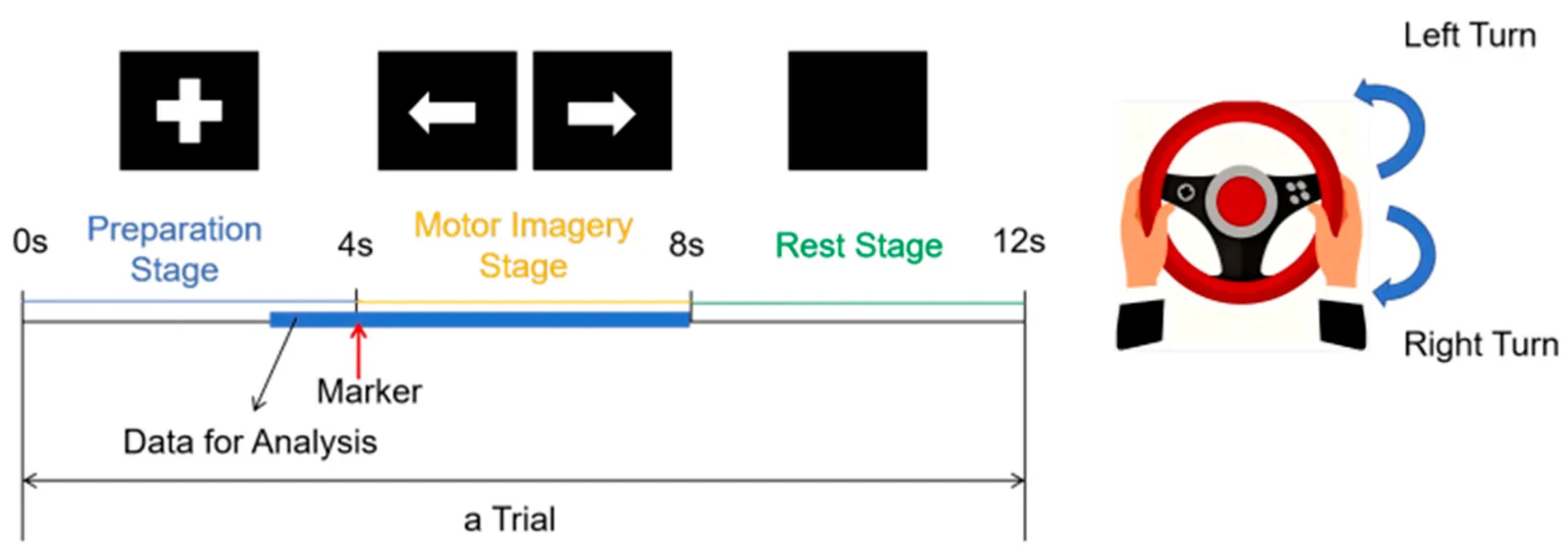
Experimental paradigm.

The experimental procedure is illustrated in Figure 1. In each trial, participants were instructed to imagine either a left-turn or right-turn steering wheel task, as cued. A single trial proceeded as follows: a white fixation cross was first presented at the center of the screen for 4 s to prompt attention and readiness. This was followed by a 4 s directional arrow cue, during which participants imagined the corresponding right-hand steering action. Subsequently, the screen turned black for a 4 s rest period. The experiment consisted of seven runs, each lasting approximately 10 min and comprising 50 randomly ordered trials (25 left-turns and 25 right-turns). A 5-min rest interval was provided between consecutive runs. Thus, each participant completed a total of 350 trials, with 175 trials for each turn direction.

### 2.2. Data Recording

**Figure 2:**
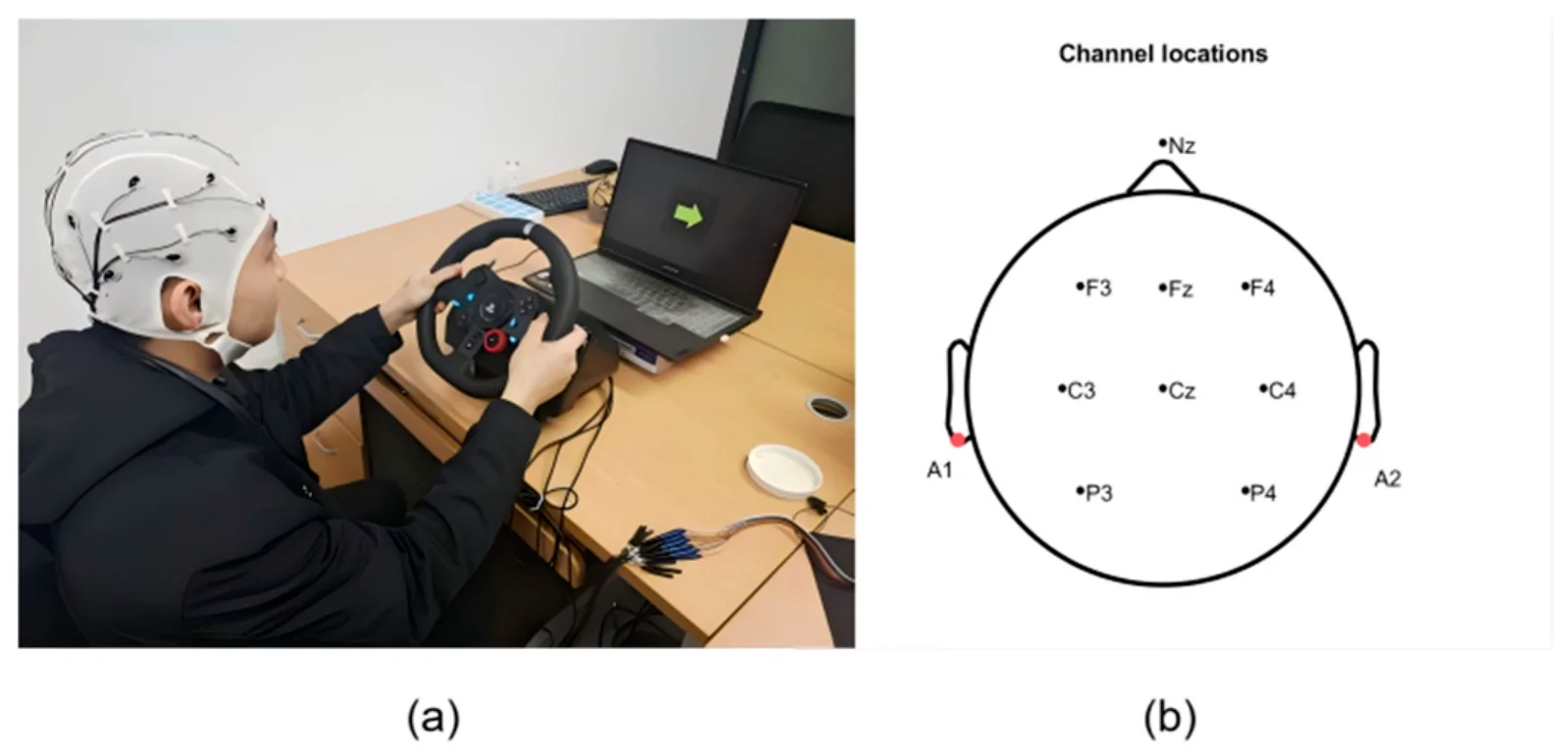
Data acquisition setup and electrode placement. (**a**) Data acquisition scene during the experiment. (**b**) Electrode placement for EEG recording.

EEG signals were recorded from participants’ scalps using an Electro-Cap System II electrode cap (Electro-Cap International, Inc., Eaton, OH, USA) connected to an OpenBCI Cyton amplifier board (OpenBCI, Inc., Brooklyn, NY, USA). Signals were sampled at 250 Hz. Electrodes were arranged according to the International 10–20 system, with eight channels over the sensorimotor cortex selected for motor-related decoding: Fz, F3, F4, Cz, C3, C4, P3, and P4. The ground and reference electrodes were positioned on the left and right earlobes (A1 and A2), respectively, as shown in Figure 2b. EEG activity associated with left- and right-hand movements was primarily measured from the C3 and C4 regions as well as the 8-electrode cluster centered on Cz, all of which carried discriminative information for decoding hand motor tasks [24]. Throughout the experiment, electrode impedance was kept below 5 KΩ. Data were recorded and stored using the OpenViBE platform.

### 2.3. Data Preprocessing

Preprocessing of EEG signals was performed in MATLAB 2025b using the EEGLAB toolbox, following the pipeline detailed below:

(1) Re-referencing: Data from all scalp electrodes were re-referenced to the Cz channel to provide a common reference.

(2) Filtering: A finite impulse response (FIR) bandpass filter (1–40 Hz) was applied to preserve movement-related neural activity while attenuating low-frequency drift and high-frequency noise (EEGLAB default Hamming window design; filter order automatically determined by the sampling rate of 250 Hz). Filtering was performed prior to epoching to avoid edge artifacts.

(3) Epoch Extraction and Baseline Correction: Epochs were extracted from −1 s to +4 s relative to each event marker. Baseline correction was then applied by subtracting the mean amplitude of the pre-stimulus interval (−1 s to 0 s) to remove DC offsets.

(4) Automatic Artifact Rejection: An automated artifact-rejection procedure was implemented using EEGLAB’s “pop_rejmenu” function, whereby trials with amplitude excursions exceeding ±100 μV were excluded. All retained trials were subsequently visually inspected to confirm or override the automated rejection labels. This step was performed before independent component analysis (ICA) to prevent large artifacts from dominating the independent component decomposition.

(5) ICA: ICA was employed to extract and remove components attributable to non-brain sources such as eye movements and muscle artifacts, further purifying the EEG signals [25]. ICA was applied to epoched data to obtain physiologically interpretable components aligned with trial-specific responses.

After preprocessing, each participant retained over 300 trials, with at least 150 trials for each of the left- and right-turn motor imagery conditions. It should be noted that among the eight recorded electrodes, Cz served exclusively as the reference electrode and was not included in subsequent feature extraction or classification; all analyses were performed on the remaining seven electrodes (F3, F4, C3, C4, P3, P4, Fz).

### 2.4. Feature Analysis

#### 2.4.1. Time–Frequency Analysis

Event-related spectral perturbation (ERSP) extends the concept of event-related desynchronization/synchronization (ERD/ERS) into the time–frequency domain, characterizing event-related changes in spectral power relative to a pre-event baseline. Through baseline correction applied at each time–frequency point, this method effectively isolates event-induced spectral modulations while minimizing influences from individual baseline differences and trial-to-trial variability.

ERSP was computed over the task period (−1 s to 4 s), with event onset defined as time 0. Given that MI predominantly modulates the mu and beta rhythms, all preprocessed signals were bandpass-filtered between 4 and 40 Hz. For the hand MI-related electrodes C3 and C4, the short-time Fourier transform was applied to derive the time–frequency power distribution during the task period. ERSP was then expressed in decibels (dB) using a trial-wise baseline normalization procedure: for each trial, the mean spectral power within the 4–40 Hz band over the baseline interval (−1 s to 0 s) was calculated as P_baseline, and the power at each subsequent time–frequency point (P_task) was converted to dB relative to this baseline via the formula $10\times {log}_{10}(P\_task/\;P\_baseline)$. This logarithmic transformation compresses the dynamic range, enhances the visibility of weaker signals, and provides a perceptually aligned scale that facilitates the identification of ERS or ERD.

#### 2.4.2. Common Spatial Pattern Analysis

Common Spatial Pattern (CSP) is designed to derive a set of spatial filters that maximize the variance of bandpass-filtered EEG signals for one class while minimizing the variance for the other class [26]. In MI-EEG analysis, CSP is commonly applied within a broad frequency range of 4–40 Hz. Although this wide band contains most MI-relevant information, it also introduces redundant components. Following the approach described in [27], spatial filters are employed to extract features associated with energy variations. Specifically, CSP seeks the spatial filter ω that optimizes the objective function [28]:

(1) $$J(\omega )=\frac{\omega^{T}X_{1}^{T}X_{1}\omega }{\omega^{T}X_{2}^{T}X_{2}\omega }=\frac{\omega^{T}C_{1}\omega }{\omega^{T}C_{2}\omega }$$

where T denotes the transpose, X is the data matrix for class *i* (with training samples as row vectors and channels as column vectors), and C is the spatial covariance matrix for class *i*, which is typically assumed to have zero mean in EEG signal processing. This assumption is generally satisfied after bandpass filtering. The optimization problem can be approached by noting that the objective function J(ω) is invariant under scaling of the filter ω; specifically, J(kω)=J(ω) for any real constant k, implying that the magnitude of ω is arbitrary. Therefore, the solution (though not unique) can be derived as follows.

Extremizing J(ω) is equivalent to extremizing $\omega^{T}C_{i}\omega$, subject to the constraint $\omega^{T}C_{i}\omega =1$, since a rescaling of ω can always be found such that $\omega^{T}C_{i}\omega =1$. Using the method of Lagrange multipliers, this constrained optimization problem reduces to extremizing the following function:

(2) $$L(\lambda ,\omega )\;=\;\omega^{T}C_{1}\omega -\lambda (\omega^{T}C_{2}\omega \;-\;1)$$

The filter at which the derivative of L with respect to ω vanishes is the one that extremizes L:

(3) $$\frac{\text{∂}L}{\text{∂}\omega }\;=\;2\omega^{T}C_{1}\;-2\lambda \omega^{T}C_{2}\;=\;0\;\Leftrightarrow C_{1}\omega =\lambda C_{2}\omega \;\Leftrightarrow {C\;}_{2}^{-1}C_{1}\omega =\lambda \omega$$

This leads to a standard eigenvalue problem. The spatial filters that extremize (1) are given by the eigenvectors of $M=C_{2}^{-1}C_{1}$, corresponding to the largest and smallest eigenvalues of this matrix. In CSP, the extracted features are the logarithmic values of the variance of EEG signals after projection onto the filter ω.

### 2.5. Baseline Models

To evaluate the decodability of left- and right-turn motor imagery, this study employed two conventional methods as baselines: CSP and FBCSP [29]. In the CSP algorithm, EEG signals were bandpass-filtered at 4–30 Hz using a 4th-order Butterworth filter, and two pairs of CSP features (four features in total) were extracted for LDA classification. The FBCSP algorithm utilized 8 sub-bands (4–36 Hz, each with a bandwidth of 4 Hz), with bandpass filtering applied to each sub-band, followed by CSP feature extraction (two pairs per sub-band, yielding 32 features in total). After feature extraction, ANOVA was performed to select the most discriminative features (up to 15 features) to reduce feature redundancy and enhance generalization capability. A nonlinear SVM with an RBF kernel was used as the classifier, with hyperparameters determined via Bayesian optimization within the training set (30 evaluations).

For deep learning methods, EEGNet [20], EEG-TCNet [30], and ATCNet [31] were selected as baseline models and compared with the proposed SMB-TCSAN model. EEGNet is a lightweight CNN that employs depthwise separable convolutions to efficiently capture spatiotemporal patterns, with a low parameter count and good generalization performance. EEG-TCNet integrates the EEGNet architecture with temporal convolutional network (TCN) capabilities, achieving a favorable balance between parameter count and accuracy. ATCNet employs multi-branch TCNs with different dilation rates, combined with dynamic convolution and multi-head attention mechanisms, to enable multi-scale temporal feature encoding. The network architecture parameters of all deep baseline models were set according to the recommended values in their original publications.

### 2.6. Proposed Architecture: Sinc Multi-Branch Temporal Convolutional Self-Attention Network

Building upon the strengths of the aforementioned benchmark algorithms, this study proposes a novel network architecture, named the Sinc Multi-Branch Temporal Convolutional Self-Attention Network (SMB-TCSAN). Specifically designed for decoding unilateral limb MI tasks, the network is well-adapted to low-density electrode configurations. It aims to adaptively extract highly discriminative frequency band and temporal features from MI-EEG signals acquired through only seven electrode channels. The lightweight architecture makes it particularly suitable for settings with a limited number of electrodes, enabling robust and accurate decoding performance even with low channel density.

**Figure 3:**
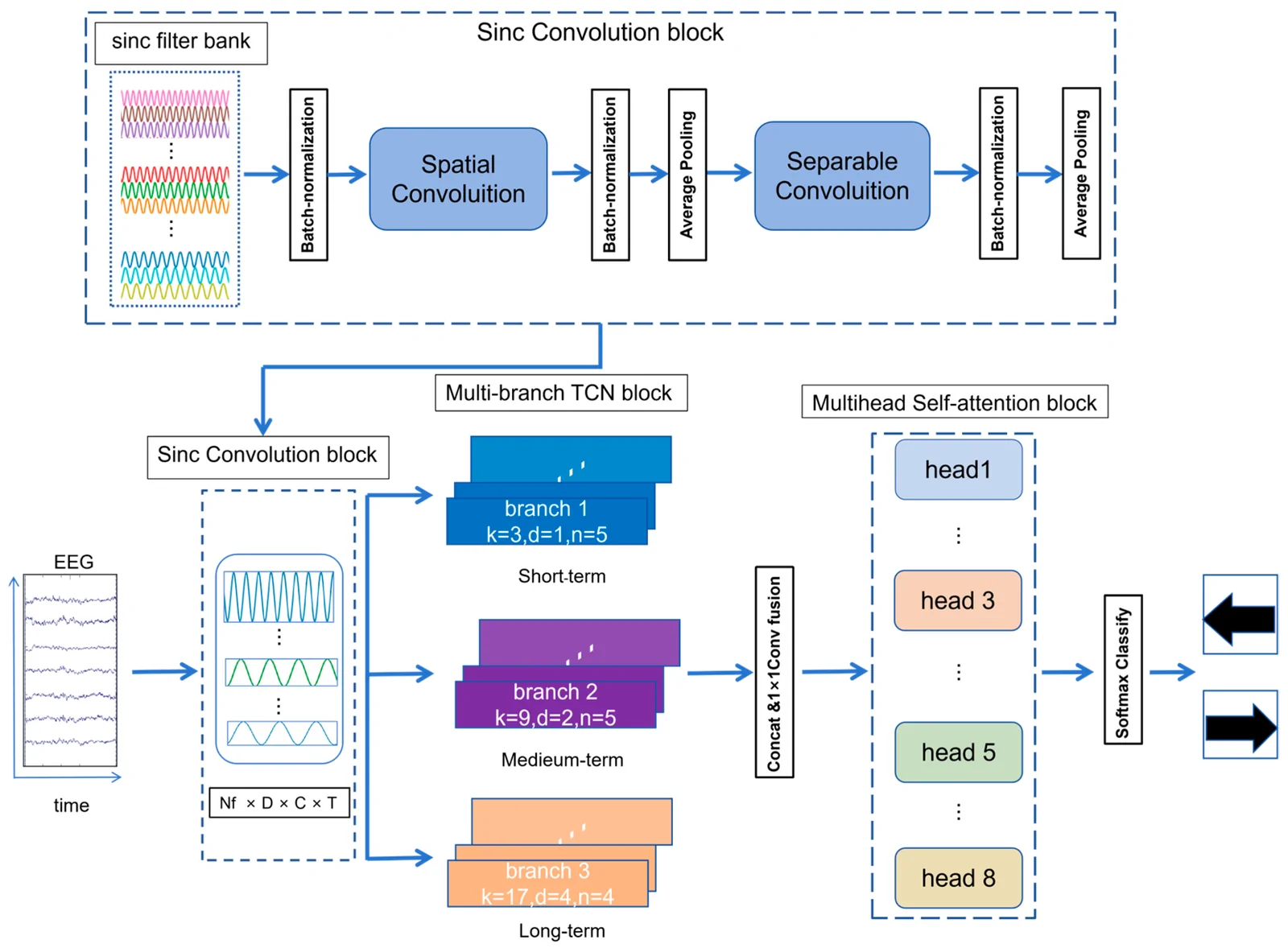
Architecture of the proposed SMB-TCSAN model.

As shown in Figure 3, the SMB-TCSAN architecture comprises three sequentially arranged core blocks. The Sinc convolution block functions as the primary feature extraction layer. It utilizes parameterized Sinc filters, instead of fixed filters, to perform adaptive bandpass filtering with minimal parameters, allowing it to automatically concentrate on task-relevant frequency bands. This approach not only improves feature interpretability but also helps prevent overfitting. Subsequently, the Multi-branch TCN block captures multi-scale dynamic features via three parallel branches, thereby expanding the model’s temporal receptive field and enhancing its capacity to model evolving neural activities. Finally, the multi-head self-attention (MSA) block conducts high-level feature integration. By computing global dependencies, it dynamically evaluates and emphasizes key temporal information, enabling the network to focus on the temporal contexts most critical for classification. Collectively, these mechanisms improve both the discriminative ability and robustness of the extracted features.

#### 2.6.1. Sinc Convolution Block

The network takes preprocessed single-trial data, denoted as $X\in R^{\left(C\times T\right)}$, along with its corresponding label $y\in \{0,1,\ldots ,N_{c}\}$. where C, T, and $N_{c}$ represent the number of channels, time points, and classes, respectively. Given that neural oscillations relevant to MI tasks are primarily concentrated in the mu and beta bands of the sensorimotor cortex, and considering the inter-subject variability in rhythmic frequencies, this block first applies a set of $N_{f}$ trainable Sinc filters to the raw signal for multi-band decomposition. This step transforms the data dimension to $\left(N_{f}\times C\times T\right)$.

Inspired by the efficiency of EEGNet, the block then sequentially executes the following steps: (i) spatial convolution with D filters to capture cross-channel topological patterns, yielding an output of size $\left(N_{f}\times D\times T\right)$; (ii) temporal downsampling via average pooling with kernel size $w_{1}$; (iii) depthwise separable convolution is used with the pointwise convolution omitted to limit parameters, thereby preserving feature dimensions; and (iv) a second temporal compression step using average pooling with kernel size $w_{2}$.

Through this pipeline, the Sinc convolution block converts the raw (C×T) EEG signal into a compact, three-dimensional tensor of size ${(N}_{f}\times D\times T^{\prime })$, where $T^{\prime }=T/(w_{1}\times w_{2})$. The resulting representation integrates multi-band spectral and spatially filtered information, providing a discriminative feature basis for subsequent temporal modeling.

#### 2.6.2. Multi-Branch Temporal Convolutional Block

The Multi-Branch Temporal Convolutional (TCN) Block acts as the core temporal feature extraction component of the proposed model, designed to capture multi-scale dynamic patterns inherent in MI-EEG signals. It accepts feature maps from the preceding Sinc convolution block, with dimensions of ${(N}_{f}\times D\times T^{\prime })$, corresponding to the temporally compressed step count. These features are first flattened along the channel dimension to the shape $\left(F_{2},T^{\prime }\right)$, with $F^{2}=N_{f}\times D$.

The block is designed based on the inherent temporal structure of MI cognitive processing: a complete trial generally includes multiple phases, such as preparation, imagery execution, and termination, each accompanied by neural activity that evolves continuously across millisecond- to second-level time scales. To model such dynamics, a parallel three-branch architecture is employed, with each branch configured using distinct convolutional kernel sizes and dilation rates to extract temporal contexts at different scales:

- Branch 1 (kernel size $K_{1}$, dilation base $d_{1}$, layers $L_{1}$) extracts local, short-term features and captures rapid neural oscillations.
- Branch 2 (kernel size $K_{2}$, dilation base $d_{2}$, layers $L_{2}$) models rhythmic modulations and correlation patterns over medium time spans.
- Branch 3 (kernel size $K_{3}$, dilation base $d_{3}$, layers $L_{3}$) captures long-range dependencies and slowly varying cognitive states.

Each branch is built as a standard temporal convolutional block, consisting sequentially of a 1D convolutional layer with weight normalization, a Chomp1d layer for trimming excess padding, a ReLU activation function, and a Dropout layer for regularization. The outputs from all three branches are concatenated along the channel dimension, followed by a 1 × 1 convolution for cross-branch feature fusion and dimensionality unification. A residual connection is also incorporated to promote stable training dynamics.

The block ultimately outputs features of shape $(48,T^{\prime }$). Through this parallel multi-scale fusion mechanism, it is capable of representing temporal dynamics ranging from fast transient events to sustained cognitive processes, thereby enhancing both the discriminability and robustness of the model in decoding MI-EEG temporal patterns.

#### 2.6.3. Multi-Head Self-Attention Block

To further extract highly discriminative information from the multi-scale temporally modeled features, the model incorporates an MSA block. This block takes as input the output features of the multi-branch TCN block, which have dimensions $\left(48,T^{\prime }\right)$. By employing a grouped multi-head self-attention mechanism, the MSA adaptively captures global dependencies across time steps, assigning higher weights to informative key phases. This effectively suppresses noise and enhances the representation of discriminative features. Moreover, the grouped multi-head mechanism partitions the input features along the channel dimension into multiple independent subspaces for parallel attention computation. This design not only reduces computational complexity but also facilitates the extraction of diverse feature correlations from multiple perspectives, such as spatiotemporal patterns and frequency band associations.

In the specific implementation, the input features are first transposed and augmented with sinusoidal positional encoding to preserve temporal order information. The query (Q), key (*K*), and value (*V*) matrices are then obtained via linear projections. The attention computation for each head is defined as follows [32]:

(4) $$\mathrm{Attention}(Q,K,V)\;=\;\mathrm{softmax}\;(\frac{QK^{T}}{\sqrt{d_{k}}})V$$

where Q, K, and V denote the query, key, and value matrices, respectively, and $d_{k}$ is the dimensionality of the key. The multi-head attention is formulated as

(5) $$MultiHead(Q,K,V)=Concat({head}_{1},\ldots ,{head}_{h})W^{O}$$

where ${head}_{i}=Attention(QW_{i}^{Q},KW_{i}^{K},{VW}_{i}^{V})$, h is the number of attention heads, and $W^{O}$ is the output projection matrix. Each head independently computes attention weights, with dropout (rate = 0.2) applied to mitigate overfitting. The outputs of all heads are concatenated along the channel dimension and fused via a learnable linear projection layer. The overall architecture adheres to the Transformer paradigm, incorporating residual connections, layer normalization, and dropout regularization to ensure training stability and generalization capability. Ultimately, the MSA block preserves the output shape of $\left(48,T^{\prime }\right)$, while its internal representations are enriched with temporal information dynamically weighted by global context. This enhances the model’s capacity to recognize key neural patterns in unilateral limb MI tasks.

The detailed parameters of the proposed SMB-TCSAN model are summarized in Table 1.

### 2.7. Training Procedure

All methods followed a unified evaluation strategy: data from each participant were divided into training and independent test sets at an 8:2 ratio, ensuring that the same trial did not appear in both sets. A within-subject 5-fold cross-validation was applied to the training set for hyperparameter selection (four folds for training, one fold for validation per fold), with no trial overlap across different sets. The feature extraction parameters of the conventional methods were estimated exclusively within the training set, and the test set was used only for projection and final evaluation.

All deep learning methods were implemented in Python 3.9 using the PyTorch library, with computations accelerated by an RTX 3080 GPU. The AdamW optimizer (initial learning rate: 0.001, weight decay: 0.01) was employed in conjunction with a OneCycleLR scheduler to dynamically adjust the learning rate (peaking at 0.01). The batch size was set to 64, with a maximum of 600 training epochs and an early stopping mechanism (patience of 20 epochs based on validation loss). The standard cross-entropy loss function was used throughout training. After training, the model parameters from the epoch with the highest validation accuracy were selected as the final model for evaluation on the independent test set.

### 2.8. Statistical Analysis

The Wilcoxon signed-rank test (paired, two-tailed) was employed to statistically compare the classification accuracies between SMB-TCSAN and each baseline algorithm, with a significance level of *α* = 0.05. The statistical analysis was conducted at the subject level (*n* = 12), where each comparison was based on the paired accuracy data of 12 participants for the two algorithms. The null hypothesis was that there was no significant difference in classification accuracy between the two algorithms.

Given that five pairwise comparisons (SMB-TCSAN vs. five baseline methods) were performed, the Benjamini–Hochberg procedure was applied for false discovery rate (FDR) control, with the FDR control level set at *q* = 0.05, and the adjusted *q*-values were reported. Effect sizes were evaluated using $r=\left|z\right|/\sqrt{n}$, where *z* is the normal approximation statistic from the Wilcoxon test. According to Cohen’s guidelines [33], r≥0.5, 0.3≤r<0.5, and r<0.3 indicate large, medium, and small effects, respectively. In addition, bootstrap 95% confidence intervals (CIs) for the median differences were computed using 10,000 resamples, where a CI that did not contain zero indicated a statistically significant difference at the 95% confidence level. All statistical tests were two-tailed and performed in MATLAB.

## 3. Results

This section presents a comprehensive evaluation of the collected steering MI dataset, including time–frequency analysis, spatial filtering, and classification performance. It first demonstrates the effectiveness of the designed steering paradigm and the discriminability of the elicited neural patterns. Furthermore, it validates the efficacy of the proposed network architecture.

### 3.1. Results of Time–Frequency Analysis

**Figure 4:**
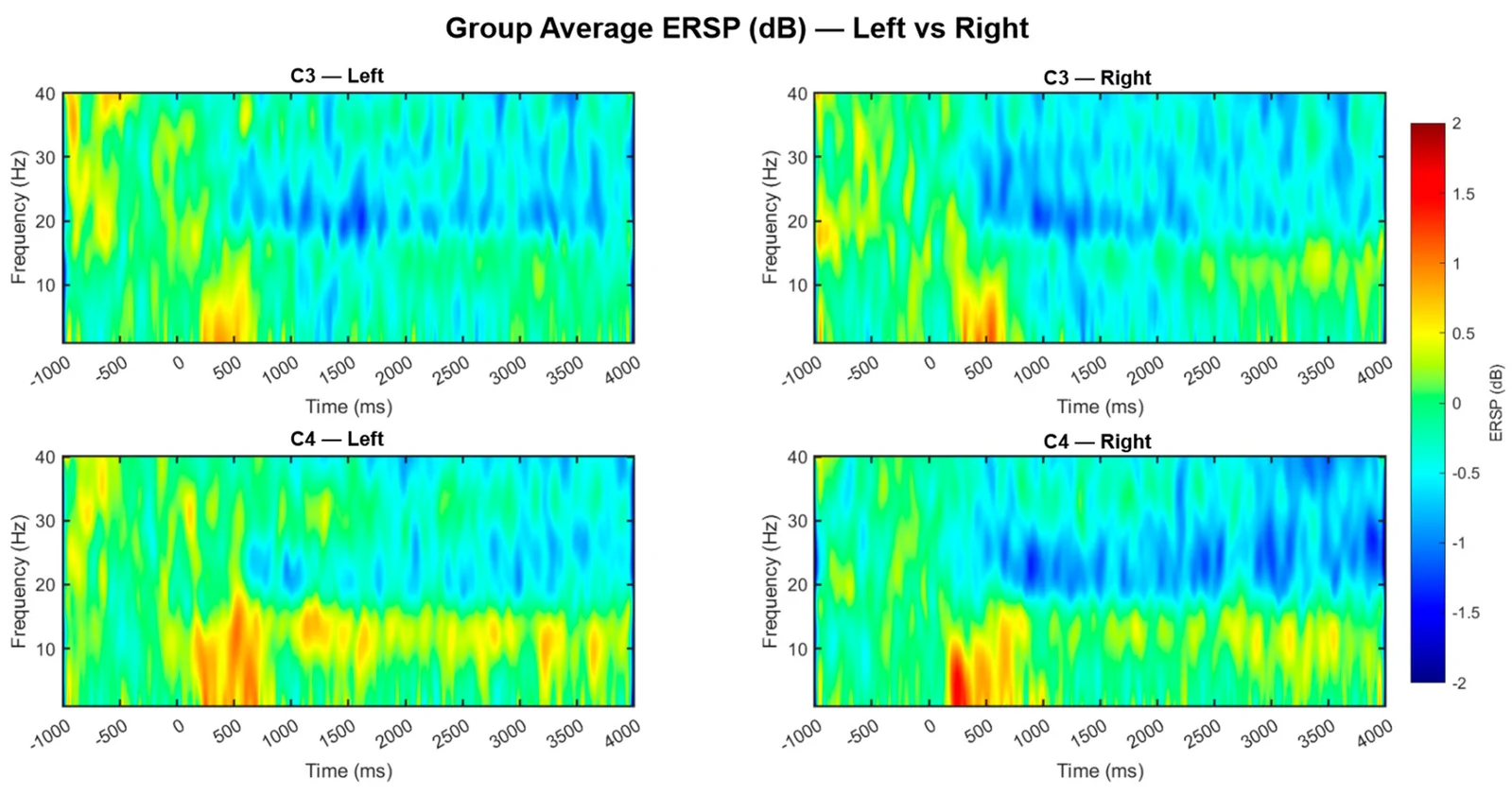
Averaged time–frequency maps of 12 participants. Blue indicates ERD. Red indicates ERS.

Figure 4 presents the group-averaged ERSP time–frequency maps at electrodes C3 and C4 across 12 participants under left-turn and right-turn motor imagery conditions. Following cue onset, spectral power in the low-frequency band (<7 Hz) increased markedly, exhibiting an ERS pattern, indicating that the cerebral cortex entered a state of motor preparation. This low-frequency enhancement was primarily concentrated in the upper-limb motor preparation phase, consistent with previous findings [34]. Subsequently, at approximately 500 ms post-cue, a clear ERD emerged, marking the onset of the motor imagery phase. In the beta band, both C3 and C4 electrodes exhibited pronounced ERD, beginning at around 500 ms post-cue and persisting until the end of the motor imagery period. In the mu band, C3 showed ERD, whereas C4 exhibited more prominent ERS, suggesting sustained sensorimotor cortex activation during the imagery process. Notably, at electrode C4 under the right-turn condition, ERSP values in the 20–30 Hz range were more negative than those in the left-turn condition, indicating a trend toward enhanced right-turn ERD.

**Figure 5:**
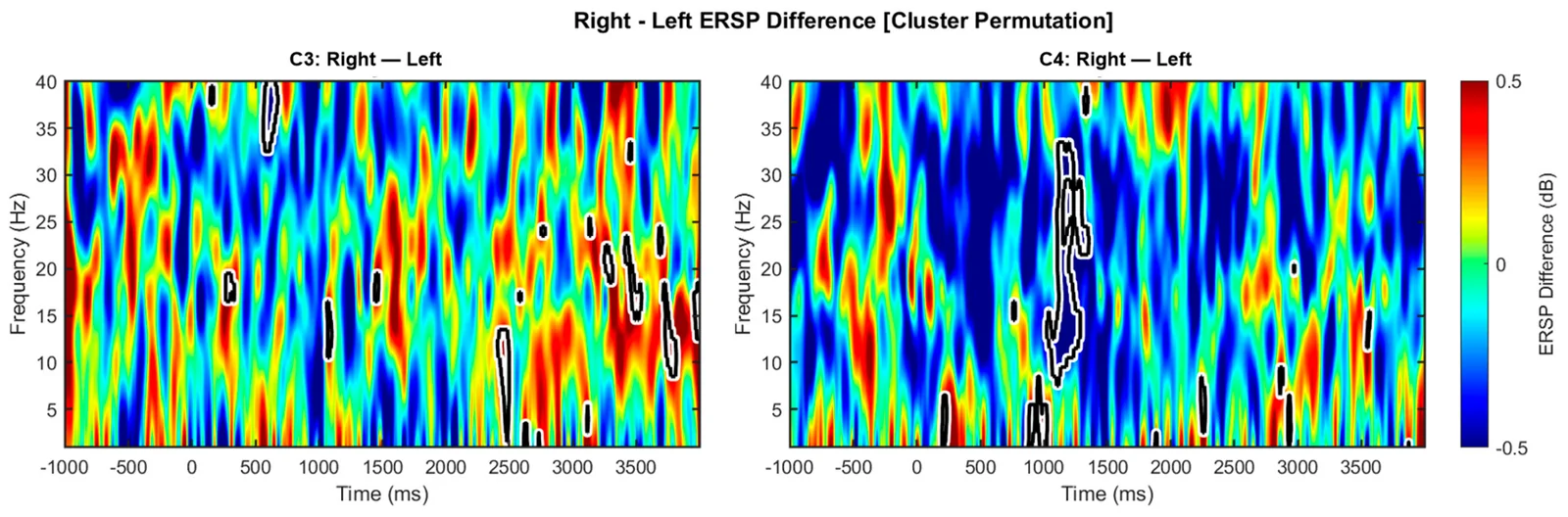
ERSP difference maps (right–left) at C3/C4. Black contours: significant clusters (*p* < 0.05, cluster-corrected, 500 perm). Red: right > left; blue: right < left.

To evaluate the statistical significance of the differences between left- and right-turn conditions in the time–frequency domain, this study employed a cluster-based permutation test [35] for statistical inference. Specifically, a paired *t*-test was performed at each time–frequency point, and neighboring points exceeding the critical threshold were connected into clusters using four-connectivity in the time–frequency plane. A null distribution of cluster mass (the sum of t-values within a cluster) was constructed via 500 random permutations, and the 95th percentile of this null distribution was used as the significance threshold (cluster-level *p* < 0.05, corrected). As shown in Figure 5, a significant time–frequency cluster was identified at electrode C4 under the right-turn condition, predominantly located in the range of approximately 1000–1500 ms and 8–35 Hz, revealing that the ERSP during right-turn trials was significantly lower than that during left-turn trials (ERSP difference < 0), indicating that right-turn imagery induced stronger sensorimotor desynchronization. No significant cluster was found at electrode C3. The significant cluster was outlined with black contours on the difference time–frequency map.

The above differences may arise from variations in action naturalness and cognitive load associated with different steering directions. For right-handed drivers, left-turn actions performed with the dominant right hand are relatively natural and may require fewer cognitive resources, thus eliciting weaker ERD. In contrast, right turns, especially large-angle turns, typically involve left-hand coordination in actual driving, which differs from the single-right-hand motor imagery paradigm employed in this study. When participants imagine right turns with only the right hand, they may need to suppress the habitual bimanual coordination pattern while also making directional judgments and spatial transformations. This additional cognitive control demand may enhance sensorimotor desynchronization, resulting in stronger ERD [36].

Individual participant analyses indicated consistent overall trends; however, the frequency bands exhibiting the most marked ERD varied across participants. For instance, Participant 1 demonstrated more pronounced ERD in the mu band, whereas Participant 2 and 3 exhibited stronger ERD in the beta band.

### 3.2. Results of Spatial Filtering Analysis

**Figure 6:**
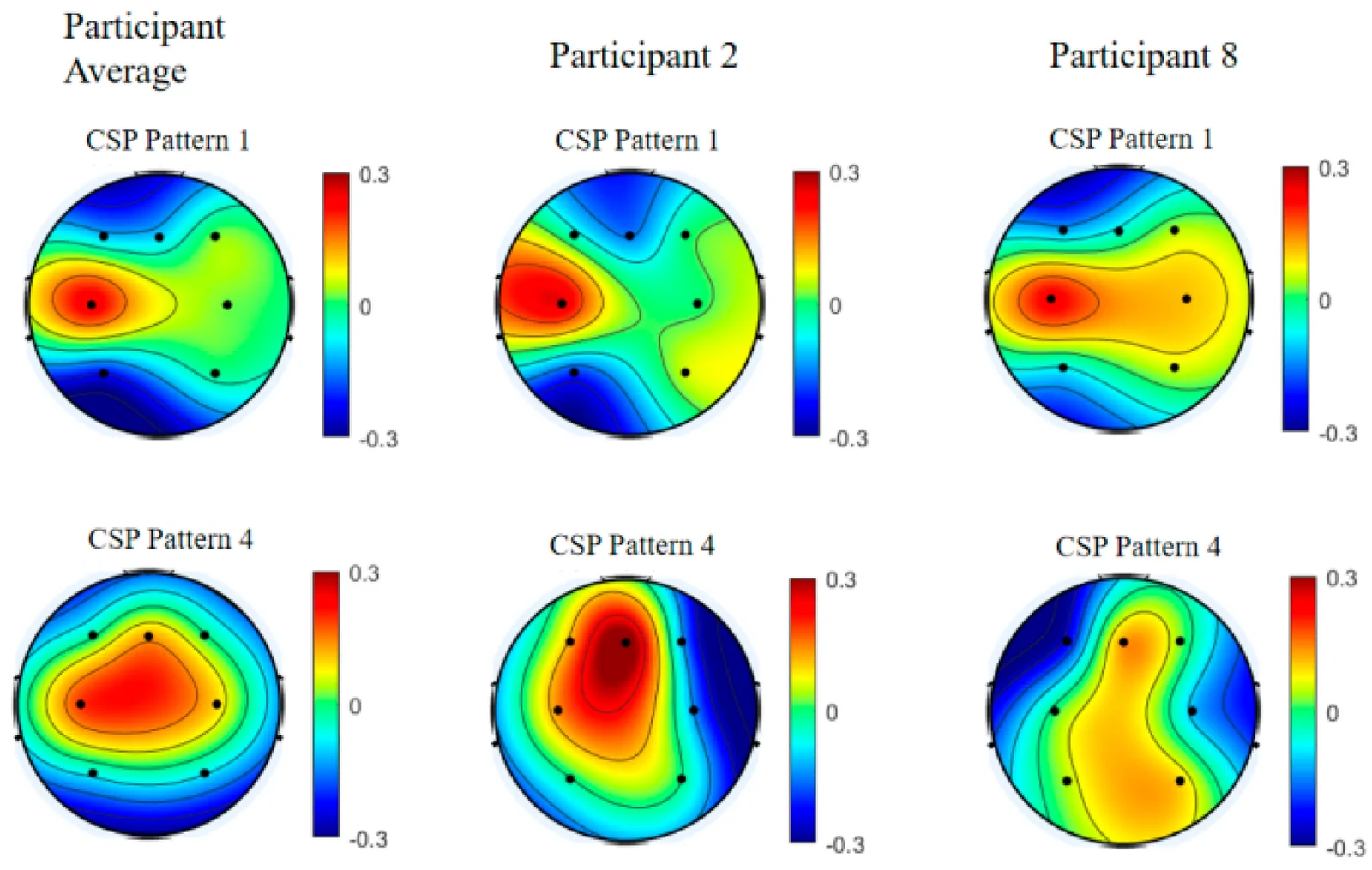
Topographical maps of the most relevant spatial pattern corresponding to the most significant feature.

Spatial patterns, which inherit the variance-separation property of spatial filters, along with their corresponding topographic maps, provide a foundation for interpreting the underlying neurophysiological mechanisms [37]. This study conducted a comprehensive analysis of the spatial patterns based on CSP feature extraction from all participants’ data. Figure 6 presents the CSP topographic maps, with the first column showing the average spatial pattern across all participants, and the second and third columns displaying individual topographic maps for the participants with the highest and lowest classification accuracy, respectively.

The CSP1 pattern in Figure 6 exhibits relatively high discriminative weights over the left central region (around C3), which is consistent with the expected contralateral sensorimotor cortex involvement during right-hand turn MI. This is reflected in the positive projections over this region in the CSP map, while the right hemisphere shows relatively lower weights. Notably, our analysis reveals task-specific discriminative spatial pattern differences, which are spatially consistent with the sensorimotor lateralization patterns observed in previous studies [38,39].

In contrast, the CSP4 pattern shows broader discriminative information distribution, involving not only the left central region but also frontal areas. This suggests that during right-turn MI tasks, participants may require greater cognitive resource engagement, which is consistent with the findings of [40] that reported frontal involvement in steering-related tasks, further indicating that the neural representations of steering intention may involve cognitive functional regions beyond the motor cortex.

The statistical significance of the average CSPs across participants was analyzed using the Wilcoxon signed-rank test, with the results presented in Table 2. Specifically, CSPs 1, 3, and 4 showed significant differences between the left-turn and right-turn MI tasks (Pattern 1: *p* = 0.0007; Pattern 3: *p* = 0.0089; Pattern 4: *p* = 0.0219), while Pattern 2 did not reach statistical significance (*p* = 0.0641). Regarding activation direction, Pattern 1 (Z = 3.380, *p* = 0.0007) and Pattern 2 (Z = 1.851, *p* = 0.0641) showed a tendency toward stronger activation during the left-turn task, whereas Pattern 3 (Z = 2.615, *p* = 0.0089) and Pattern 4 (Z = 2.293, *p* = 0.0219) exhibited significantly stronger activation during the right-turn task.

### 3.3. Steering Intention Decoding Performance

**Figure 7:**
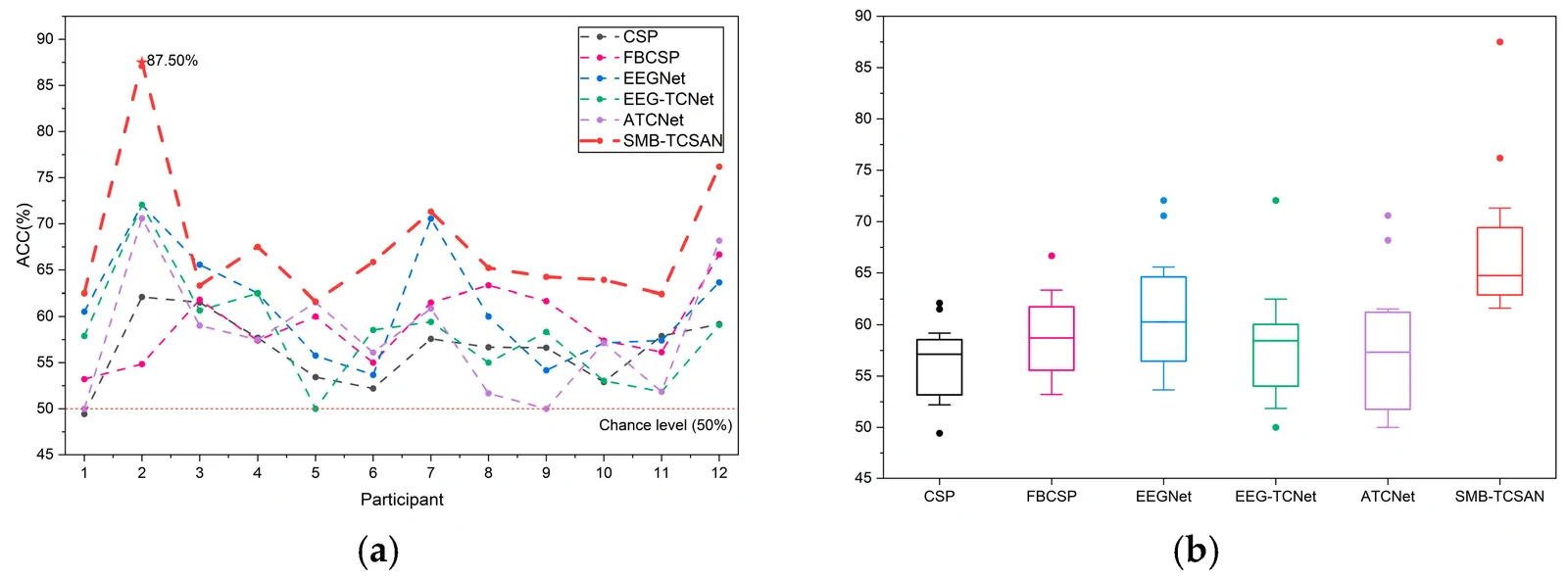
Classification accuracy for each participant under the 5-fold CV setting is presented: (**a**) per-participant accuracy curves, with 50% chance level (red dashed) and peak accuracy (87.5%, Participant 2, asterisk), (**b**) across-participant boxplots (colors as in (**a**); boxes = IQR, lines = median, dots = outliers).

To evaluate the decoding performance of the proposed steering motor imagery paradigm, five advanced algorithms in the MI-BCI field, including CSP, FBCSP, EEGNet, EEG-TCNet, and ATCNet, were selected as baseline methods for comparison with the proposed SMB-TCSAN. The classification accuracies of all algorithms are summarized in Table 3, and Figure 7 presents the individual performance distribution of the six algorithms across the 12 participants.

As shown in Figure 7a, all algorithms effectively decoded steering intention, although notable individual differences and performance fluctuations were observed across participants. Notably, all participants achieved classification accuracies significantly above the chance level (50%) for the proposed SMB-TCSAN model, confirming that the observed performance is consistent rather than driven by a small subset of high-performing individuals. Among the five baseline algorithms, EEGNet achieved the highest mean accuracy of 61.08%, whereas the proposed SMB-TCSAN attained an average accuracy of 66.80%, corresponding to a 5.72% improvement over EEGNet. Relative to the other baselines, SMB-TCSAN outperformed CSP by 10.36%, FBCSP by 7.72%, EEG-TCNet by 8.60%, and ATCNet by 8.93%. Moreover, SMB-TCSAN consistently surpassed the best baseline for the majority of participants, with Participant 2 achieving the highest individual decoding accuracy of 87.50%.

The box-and-whisker plot in Figure 7b further illustrates the performance differences between the various algorithms. Both the median and interquartile range (IQR) of SMB-TCSAN are higher than those of all other compared algorithms, and its box height (IQR) is smaller. This indicates that the model maintains high decoding performance with minimal variation across different participants. In contrast, CSP yields the lowest overall performance, with a median of 57.12%, whereas FBCSP performs slightly better. EEGNet, EEG-TCNet, and ATCNet exhibit intermediate distributions, but their overall performance still falls short of that of SMB-TCSAN.

To further quantify performance differences, a paired two-sided Wilcoxon signed-rank test was used to analyze the classification accuracy of the 12 participants. Multiple comparisons were corrected using the Benjamini–Hochberg FDR (*q* = 0.05), whilst the effect size *r* and 95% CI were calculated using the bootstrap method (10,000 resamples). The test results are presented in Table 3. The statistical results indicate that SMB-TCSAN showed statistically significant differences compared with all five baseline algorithms (all *q* < 0.05 after FDR correction). The median accuracy improvements ranged from 5.42% to 10.42%, with the largest improvement of 10.42% over CSP and a 5.42% improvement over EEGNet. The effect sizes *r* for all comparisons were greater than 0.5 (0.793~0.883), indicating a strong effect. And all bootstrap 95% confidence intervals were entirely positive, further confirming that SMB-TCSAN consistently outperforms the compared baseline algorithms in decoding performance.

Combining the individual accuracy curves and box-and-whisker plots in Figure 7 with the statistical test results in Table 3, it can be concluded that, in the driving steering imagery decoding task, the proposed SMB-TCSAN model achieves higher and statistically significant classification accuracy compared to traditional spatial filtering algorithms and existing deep learning baseline models.

### 3.4. Ablation Study

**Figure 8:**
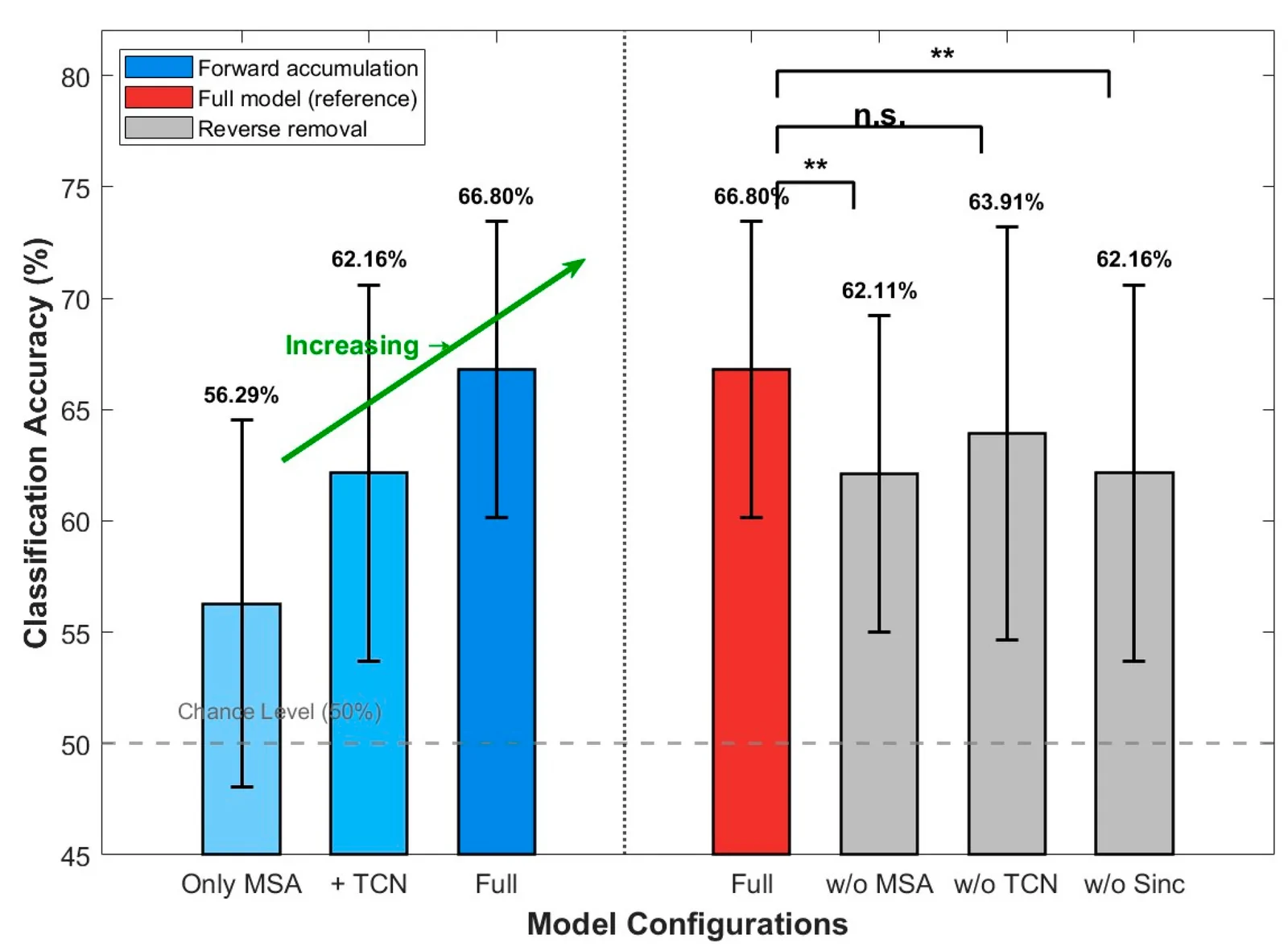
Bidirectional ablation analysis of the SMB-TCSAN model. The forward accumulation (**left**) and leave-one-out removal (**right**) strategies jointly validate the contribution of each module. ** indicates a significant difference (*p* < 0.01), and n.s. indicates no significant difference *(p* > 0.05).

To dissect the individual contribution of each constituent module in the proposed SMB-TCSAN model, we performed a systematic ablation analysis employing two complementary strategies: forward accumulation and leave-one-out removal. Figure 8 depicts the performance trajectories under these two configurations, while Table 4 summarizes the corresponding quantitative results.

In the forward accumulation path, using only the MSA block to extract temporal features via self-attention, yielded a moderate accuracy of 56.29% ± 8.23%. Incorporating the multi-branch TCN to capture multi-scale temporal features significantly boosted the performance to 62.16% ± 8.44%, with a gain of 5.87%. Finally, adding the Sinc convolution to embed frequency-specific priors completed the full model, which achieved the best accuracy of 66.80% ± 6.64%, further improving by 4.64%. This progressive improvement consistently confirms that each module positively contributes to the decoding task.

Conversely, in the leave-one-out removal path, ablating the MSA from the full model caused the largest performance drop of 4.69% (from 66.80% to 62.11% ± 7.11%, *p* = 0.009), highlighting its critical role in capturing long-range dependencies. Removing the Sinc convolution resulted in a drop of 4.64% (to 62.16% ± 8.44%, *p* = 0.005), validating the importance of frequency-specific feature extraction. In contrast, ablating the multi-branch TCN yielded a moderate drop of 2.89% (to 63.91% ± 9.27%, *p* = 0.052), which, although not statistically significant at the 0.05 level, still suggests a consistent degradation trend, indicating that multi-scale temporal modeling provides complementary contributions.

Notably, although MSA alone performed poorly (56.29% ± 8.23%), its removal from the full model induced the largest degradation, revealing a strong synergistic effect. This apparent paradox is explained by the fact that the MSA module benefits from the rich spatiotemporal representations extracted by the convolutional front-end (Sinc and TCN) to effectively model global dependencies; without these representations, its attention capacity is underutilized. Together, these three modules non-redundantly address different aspects of EEG variability, and their integration yields the most robust and accurate classification performance.

## 4. Discussion

### 4.1. Innovative Steering Motor Imagery Paradigm

The ERD/ERS features elicited by multi-limb motor tasks exhibit distinct spatial distribution patterns in the brain, aligning closely with the corresponding cortical motor areas. Consequently, traditional MI-BCIs typically focus on recognizing multi-limb MI patterns. However, applying such paradigms to vehicle control conflicts with drivers’ actual operating habits may impose additional cognitive load. Meanwhile, research on EEG signals during unilateral limb movements still faces multiple challenges, including high action complexity, strong similarity between different movements, and significant spatial overlap of neural activities [41].

To address these challenges, this study designed a right-hand steering MI paradigm that conforms to drivers’ operational habits. Through time–frequency analysis, spatial pattern analysis, and classification accuracy evaluation, significant differences in neural representations between left- and right-turn were revealed. Compared with traditional multi-limb paradigms, the proposed paradigm focuses on unilateral limb movements and aligns more closely with driving operations at the action level, offering a new perspective for the design of driving-oriented BCI paradigms.

Although distinguishing multiple tasks from unilateral limb EEG signals remains technically challenging, this study preliminarily validates its feasibility. Furthermore, this new paradigm provides a new avenue for future research to explore the complex relationship between EEG signals and steering intention. The steering direction dataset constructed based on this paradigm can support the development of non-invasive neural signal algorithms for motion direction decoding, thereby expanding the boundaries of BCI paradigms and enriching the dimensionality of MI-based control commands.

It should be noted that this study used only eight electrodes, motivated primarily by the portability and cost-effectiveness of the minimal OpenBCI configuration, with the goal of exploring low-cost BCI feasibility. While this low-density montage inevitably compromises spatial resolution and source localization relative to high-density EEG, the 66.80% accuracy achieved offers preliminary validation of its practicality.

### 4.2. Performance of Conventional and State-of-the-Art Decoding Architectures

To better design decoding algorithms suitable for the steering direction dataset, the baseline algorithms were further compared from the perspective of network architecture. As shown in Figure 7, EEGNet emerged as the top performer, achieving an average classification accuracy of 61.08%. EEGNet employs depthwise separable convolutions to automatically extract spatiotemporal frequency features without requiring manually designed filters. With only around 3000 parameters, the model is particularly well-suited to the relatively small sample size of this study and exhibits strong resistance to overfitting.

In contrast, the strength of FBCSP lies in its small standard deviation, indicating more balanced decoding performance across participants. By applying multi-band filtering, this method effectively localizes the mu and beta rhythm bands most relevant to MI, offering high interpretability and computational efficiency. Notably, time–frequency analysis revealed that the specific frequency bands exhibiting ERD/ERS during MI tasks vary among individuals. Therefore, the filter bank design, which finely partitions the frequency spectrum, helps extract the most discriminative frequency information from EEG signals, thereby enhancing feature extraction and improving model performance.

Furthermore, EEG-TCNet also delivered competitive classification results on this dataset, as shown in Table 3. Unlike conventional CNNs, TCNs are specifically tailored to sequential data. The strong performance of EEG-TCNet suggests that the steering direction dataset contains informative patterns in both temporal and spectral domains. This insight provides a useful foundation for designing lightweight and effective decoders for similar data in future work.

Building upon the strengths of EEGNet, our proposed SMB-TCSAN further improves the decoding performance. The Sinc convolution block adaptively learns subject-specific frequency filters, which is crucial given the inter-individual variability in ERD frequency bands observed in our time–frequency analysis (Section 3.1). The multi-branch TCN block, with its parallel dilated convolutions, captures temporal dynamics across multiple scales, from rapid neural oscillations to sustained imagery states, which is particularly beneficial for the 4 s imagery period in our paradigm. The subsequent multi-head self-attention mechanism then reweights the temporal features to suppress irrelevant noise and highlight the most discriminative phases of the imagery process. Together, these three modules non-redundantly address different aspects of EEG variability, and their integration yields the most robust and accurate classification performance. This hierarchical design, tailored to the characteristics of steering MI, explains the 5.72% improvement over EEGNet. The proposed SMB-TCSAN contains approximately 0.5 million trainable parameters, with an inference time of 4.25 ms per trial on an RTX 3080 GPU, which is sufficiently low for real-time BCI applications.

### 4.3. Directional Discriminative Information in EEG

Time–frequency analysis revealed that, during motor imagery, electrode C3 exhibited pronounced ERD in both the mu and beta bands, while electrode C4 showed ERD in the beta band but ERS was more prominent in the mu band. A significant difference between left- and right-turn conditions was observed at electrode C4 in the beta band: the right-turn condition induced stronger ERD than the left-turn condition, predominantly in the range of approximately 1000–1500 ms and 8–35 Hz (cluster-level *p* < 0.05, corrected). This result indicates that right-turn imagery elicited stronger sensorimotor desynchronization during the early execution phase of motor imagery.

Spatial pattern analysis indicated that the discriminative spatial projections of CSP were predominantly concentrated over the left central region during left-turn imagery, whereas right-turn imagery additionally involved frontal regions. Although CSP results themselves should not be directly equated with cortical activation, the observed frontal and central distributions are consistent with sensorimotor and prefrontal activation regions reported in source-localization studies during right-turn processes. Combined with the differences observed in the beta band at electrode C4 in the time–frequency analysis, the two steering directions exhibit distinguishable neural activation patters, providing neurophysiological plausibility for the proposed paradigm.

Individual analysis revealed that the most prominent ERD frequency bands varied across participants, with some showing stronger ERD in the mu band and others in the beta band, highlighting the importance of incorporating adaptive frequency filtering in decoding models. Based on these observations, it is hypothesized that EEG signals evoked during steering-related MI are jointly influenced by individual driving habits and driving experience. Classification performance further showed that participants with greater driving experience exhibited clearer and more focused mental simulation during motor imagery, providing preliminary behavioral support for the association between the proposed paradigm and real driving behavior.

In summary, this study demonstrates the capability of EEG signals to decode directional steering information and offers valuable insights into the future design of motor imagery-based paradigms and the optimization of control–command strategies for driving assistive BCI systems.

### 4.4. Limitations and Directions for Future Research

Several limitations of this study should be acknowledged. First, the sample size was relatively small (N = 12), and the participants were predominantly healthy, right-handed young university students, which limits the generalizability of the findings to broader populations, including older adults, left-handed individuals, and clinical patients. Second, the experiment was conducted in a seated laboratory setting with visual arrow cues, which differs substantially from the complex conditions encountered in real-world driving, such as vibrations, noise, and dynamic traffic scenarios. Moreover, because the visual cues remained present throughout the entire 4 s imagery period, the decoded signals reflect a composite task state involving both sustained visual processing and motor imagery, rather than purely MI-related activity. Third, the within-subject evaluation framework adopted in this study reflects subject-dependent decoding performance; the cross-session and cross-subject generalization capabilities of the SMB-TCSAN model have not yet been systematically validated [42], and its effectiveness and robustness in real-world environments require further investigation. Fourth, this study employed only eight electrodes, a choice primarily motivated by portability and cost-effectiveness. However, this low-density montage also limits spatial resolution, making it difficult to precisely localize cortical sources and thereby imposing a theoretical ceiling on decoding performance. The 66.80% accuracy achieved in this study provides preliminary evidence for the feasibility of this configuration, though it still falls short of the performance typically achieved by high-density EEG in comparable studies.

Future research could be pursued in the following directions. First, a larger and more diverse participant cohort will be recruited, covering a broader range of ages, genders, handedness, and occupational backgrounds. This will allow validation of the current paradigm and model across varied populations while also exploring its potential applicability in rehabilitation medicine. Second, to address cross-trial and cross-subject variability, future work will explore domain adaptation and transfer learning techniques to mitigate the impact of individual differences and trial-specific effects on decoding performance. Third, driving simulators and eventually real-vehicle platforms will be introduced to establish experimental environments that more closely resemble actual driving conditions. A brief cue followed by a pure imagery period will be adopted to isolate the MI component from visual processing. Fourth, decoding will move beyond binary left/right steering classification to include multidimensional steering parameters (e.g., bimanual coordination, speed, and angle), enabling finer-grained steering intention decoding. On this basis, the external device command set will be further extended to provide a more comprehensive technical framework for driving assistive BCI systems. In addition, future work may consider higher-density electrode configurations (e.g., 16 or 32 channels) to improve spatial resolution, enable more precise cortical source localization, and further elucidate the neural mechanisms underlying steering intention.

## 5. Conclusions

This study designs a MI-BCI experimental paradigm for driving scenarios, mapping left- and right-turn imagery actions to corresponding steering intention, thereby providing an experimental foundation for exploring MI research that more closely approximates real driving intention. The feasibility of this paradigm in eliciting steering direction-related EEG features was validated through data acquisition and analysis of 12 participants.

Building upon this foundation, the study systematically analyzes the EEG features elicited by the paradigm from both time–frequency and spatial perspectives. Time–frequency analysis revealed that electrode C3 exhibited pronounced ERD in both the mu and beta bands, while electrode C4 showed ERD in the beta band but ERS was more prominent in the mu band. A significant difference between left- and right-turn conditions was observed at electrode C4 in the beta band (approximately 1000–1500 ms and 8–35 Hz, cluster-level *p* < 0.05, corrected). Spatial pattern analysis further revealed that the discriminative spatial projections of CSP were predominantly concentrated over the left central region during left-turn imagery, whereas right-turn imagery additionally involved frontal regions.

In terms of decoding performance, baseline methods including CSP, FBCSP, EEGNet, EEG-TCNet, and ATCNet were employed to classify the EEG data under this paradigm, preliminarily validating the separability of left- and right-turn tasks. On this basis, the study further proposes the SMB-TCSAN network model, which integrates Sinc convolution, multi-branch temporal convolution, and multi-head self-attention mechanisms. The model achieved an average classification accuracy of 66.80% on the steering direction dataset, outperforming all baseline methods, with the highest individual accuracy reaching 87.5%. In addition, individual difference analysis revealed that participants with more consistent imagery strategies achieved higher classification accuracy, suggesting that individual differences in imagery strategy may affect decoding performance.

These results indicate that the proposed steering motor imagery paradigm can elicit directionally discriminable EEG features, and that the SMB-TCSAN model demonstrates promising potential in decoding such features. This study provides preliminary experimental evidence for EEG-based steering intention decoding and offers a reference baseline for future research on motor imagery brain–computer interfaces in driving scenarios.

## Figures and Tables

**Table 1:** Network parameter configuration.

Module	Parameter	Value
Input data	C/T/$N_{c}$	7/1000/2
Sinc Convolutional block	$N_{f}$/D	18/2
	$w_{1}$/$w_{2}$	4/8
	$T^{\prime }$	$T/(w_{1}\times w_{2})=31$
Multi-branch TCN block	$F_{2}$	$N_{f}\times D$ = 36
	$K_{1}$/$d_{1}$/$L_{1}$	3/1/5
	$K_{2}$/$d_{2}$/$L_{2}$	9/2/5
	$K_{3}$/$d_{3}$/$L_{3}$	17/4/4
MSA block	h/$d_{k}$	8/6
	Output shape	(48,31)

**Table 2:** Significance analysis results of participant-averaged CSPs.

Metric	CSP 1	CSP 2	CSP 3	CSP 4
Z value	3.380	1.851	2.615	2.293
*p*-value	0.0007	0.0641	0.0089	0.0219
Direction of difference	Left > Right	Left > Right	Right > Left	Right > Left

**Table 3:** Classification accuracy and paired comparisons.

Algorithm	Mean (%)	Std (%)	Median Difference (%)	FDR *q*-Value	Effect Size r	95% CI
CSP	56.44	3.81	10.42	0.0024	0.883	[5.59, 13.69]
FBCSP [29]	59.08	4.05	7.92	0.0008	0.793	[1.60, 9.94]
EEGNet [20]	61.08	6.04	5.42	0.0008	0.793	[1.59, 9.76]
EEG-TCNet [30]	58.20	5.75	8.87	0.0012	0.883	[4.96, 11.26]
ATCNet [31]	57.63	6.34	10.22	0.0008	0.883	[8.17, 11.39]
SMB-TCSAN	66.80	6.64	-	-	-	-

Table notes: *p*-values were corrected using the Benjamini–Hochberg FDR (*q* = 0.05). Effect size $r=\left|z\right|/\sqrt{n}$; *r* ≥ 0.5 indicates a strong effect. Confidence intervals (CIs) were obtained via the bootstrap method (10,000 resamples).

**Table 4:** Ablation study results.

Model	Sinc	MB-TCN	MSA	Acc (%) Mean ± Std	ΔAcc (w.r.t Baseline)
Baseline (Full Model)	√	√	√	66.80 ± 6.64	0
w/o MSA	√	√	×	62.11 ± 7.11	–4.69
w/o Sinc	×	√	√	62.16 ± 8.44	–4.64
w/o MB-TCN	√	×	√	63.91 ± 9.27	–2.89
Only MSA (Stand-alone)	×	×	√	56.29 ± 8.23	–10.51

Table footnote: Values are mean ± standard deviation over 12 participants. ΔAcc denotes the difference from the baseline. “√” denotes presence; “×” denotes removal.

## Data Availability

The data presented in this study are available on request from the corresponding author due to privacy and ethical restrictions. The data contain sensitive participant information and cannot be shared.
